# Challenges facing HIV treatment in Guinea-Bissau: the benefits of international research collaborations

**DOI:** 10.2471/BLT.14.135749

**Published:** 2014-09-26

**Authors:** Sanne Jespersen, Bo Langhoff Hønge, Inés Oliveira, Candida Medina, David da Silva Té, Faustino Gomes Correia, Zacarias José da Silva, Christian Erikstrup, Lars Østergaard, Alex Lund Laursen, Christian Wejse, on behalf of the, Bissau HIV cohort study group

**Affiliations:** a Projecto de Saúde de Bandim, INDEPTH Network, Apertado 861, 1004 Bissau Codex, Guinea-Bissau.; b National HIV Programme, Ministry of Health, Bissau, Guinea-Bissau.; c National Public Health Laboratory, Bissau, Guinea-Bissau.; d Department of Clinical Immunology, Aarhus University Hospital, Denmark.; e Department of Infectious Diseases, Aarhus University Hospital, Aarhus, Denmark.; f GloHAU, Center for Global Health, Aarhus University, Denmark.

## Abstract

**Problem:**

The introduction of antiretroviral therapy (ART) for HIV infection in sub-Saharan Africa has improved the quality of life of millions of people and reduced mortality. However, substantial problems with the infrastructure for ART delivery remain.

**Approach:**

Clinicians and researchers at an HIV clinic in Guinea-Bissau identified problems with the delivery of ART by establishing a clinical database and by collaborating with international researchers.

**Local setting:**

The Bissau HIV cohort study group was established in 2007 as a collaboration between local HIV physicians and international HIV researchers. Patients were recruited from the HIV clinic at the country’s main hospital in the capital Bissau.

**Relevant changes:**

Between 2005 and 2013, 5514 HIV-positive patients were treated at the clinic. Working together, local health-care workers and international researchers identified the main problems affecting ART delivery: inadequate drug supply; loss of patients to follow-up; and inadequate laboratory services. Solutions to these problems were devised. The collaborations encouraged local physicians to start their own research projects to find possible solutions to problems at the clinic.

**Lessons learnt:**

The HIV clinic in Bissau faced numerous obstacles in delivering ART at a sufficiently high quality and patients’ lives were put in jeopardy. The effectiveness of ART could be enhanced by delivering it as part of an international research collaboration since such collaborations can help identify problems, find solutions and increase the capacity of the health-care system.

## Résumé

**Problème:**

L'introduction de la thérapie antirétrovirale (TAR) pour traiter la contamination par le VIH en Afrique subsaharienne a amélioré la qualité de vie de millions de personnes et réduit la mortalité. Cependant, l'infrastructure de distribution de la TAR reste très problématique.

**Approche:**

Les cliniciens et les chercheurs d'une clinique de Guinée-Bissau ont identifié des problèmes avec la distribution de la TAR en créant une base de données clinique et en collaborant avec des chercheurs internationaux.

**Environnement local:**

Le groupe d'étude de la cohorte VIH de Bissau a été créé en 2007 comme une collaboration entre des médecins locaux traitant le VIH et des chercheurs internationaux étudiant le VIH. Les patients ont été recrutés à la clinique VIH située dans l'hôpital principal du pays à Bissau, la capitale.

**Changements significatifs:**

Entre 2005 et 2013, 5514 patients séropositifs ont été traités à la clinique. En travaillant ensemble, les personnels de santé locaux et les chercheurs internationaux ont identifié les problèmes principaux qui affectaient la distribution de la TAR: un approvisionnement inapproprié des médicaments; des patients perdus de vue et des services de laboratoire inadéquats. Des solutions visant à résoudre ces problèmes ont été envisagées. Les collaborations ont encouragé les médecins locaux à lancer leurs propres projets de recherche afin de trouver des solutions possibles aux problèmes rencontrés à la clinique.

**Leçons tirées:**

La clinique VIH de Bissau a dû faire face à de nombreux obstacles pour distribuer une TAR d'une qualité suffisamment élevée, et les vies des patients ont été mises en péril. L'efficacité de la TAR pourrait être augmentée en la distribuant dans le cadre de collaborations de recherche internationale, puisque ces collaborations peuvent aider à identifier les problèmes, à trouver des solutions et à augmenter la capacité du système de santé.

## Resumen

**Situación:**

La introducción de la terapia antirretroviral (TARV) para tratar la infección del VIH en el África subsahariana ha mejorado la calidad de vida de millones de personas y ha reducido la mortalidad. No obstante, siguen existiendo problemas considerables relacionados con la infraestructura para el suministro de la terapia antirretroviral.

**Enfoque:**

En una clínica del VIH de Guinea-Bissau, los médicos y los investigadores detectaron problemas en el suministro de la terapia antirretroviral mediante la creación de una base de datos y la colaboración con investigadores internacionales.

**Marco regional:**

El grupo del estudio de cohorte****para el VIH de Bissau se creó en 2007, fruto de la colaboración entre los médicos del VIH locales y los investigadores del VIH internacionales. Se seleccionó a los pacientes de la clínica de VIH en el hospital principal del país de la capital Bissau.

**Cambios importantes:**

Entre 2005 y 2013, se trató a 5514 pacientes seropositivos para el VIH en la clínica. Trabajando en equipo, el personal sanitario local y los investigadores internacionales identificaron los problemas principales que repercuten en el suministro de la terapia antirretroviral: el suministro inadecuado de medicamentos; la pérdida de pacientes durante el seguimiento y los servicios de laboratorio inadecuados. Se diseñaron soluciones para estos problemas. Las colaboraciones animaron a los médicos locales a emprender sus propios proyectos de investigación a fin de hallar soluciones a los problemas en la clínica.

**Lecciones aprendidas:**

La clínica del VIH de Bissau afrontó diversos obstáculos en el suministro de una terapia antirretroviral de calidad suficientemente alta y se pusieron en riesgo las vidas de los pacientes. Es posible aumentar la eficacia de la terapia antirretroviral si esta se suministra como parte de la colaboración de una investigación internacional, ya que este tipo de colaboraciones pueden ayudar a identificar los problemas, hallar soluciones y aumentar la capacidad del sistema sanitario.

## ملخص

**المشكلة:**

أدى استخدام العلاج بمضادات الفيروسات القهقرية لعدوى فيروس العوز المناعي البشري في أفريقيا جنوب الصحراء الكبرى إلى تحسين جودة حياة ملايين الأشخاص وتقليل معدل الوفيات. إلا أنه ما زالت هناك مشكلات أساسية تتعلق بالبنية الأساسية لإيتاء العلاج بمضادات الفيروسات القهقرية.

**الأسلوب:**

قام الأخصائيون السريريون والباحثون في عيادة لفيروس العوز المناعي البشري في غينيا-بيساو بتحديد المشكلات التي تتعلق بإيتاء العلاج بمضادات الفيروسات القهقرية عن طريق إنشاء قاعدة بيانات سريرية والتعاون مع الباحثين الدوليين.

**المواقع المحلية:**

تم إنشاء فريق الدراسة الأترابية لفيروس العوز المناعي البشري في بيساو عام 2007 كوجه من أوجه التعاون بين أطباء فيروس العوز المناعي البشري المحليين وباحثي فيروس العوز المناعي البشري الدوليين. وتم الاستعانة بالمرضى من عيادة فيروس العوز المناعي البشري في المستشفى الرئيسي بالبلد في العاصمة بيساو.

**التغيّرات ذات الصلة:**

تم علاج 5514 مريضاً إيجابياً لفيروس العوز المناعي البشري في العيادة. وقام العاملون المحليون في مجال الرعاية الصحية والباحثون الدوليون معاً بتحديد المشكلات الرئيسية التي تؤثر على إيتاء العلاج بمضادات الفيروسات القهقرية، وهي: عدم كفاية إمدادات الدواء؛ وفقدان المرضى الذين يتعين متابعتهم؛ وعدم كفاية الخدمات المختبرية. وتم استحداث حلول لهذه المشكلات. وشجعت أوجه التعاون الأطباء المحليين على بدء مشاريعهم البحثية في شكل حلول ممكنة للمشكلات في العيادة.

**الدروس المستفادة:**

واجهت عيادة فيروس العوز المناعي البشري في بيساو عدة عقبات في إيتاء العلاج بمضادات الفيروسات القهقرية بجودة عالية بالقدر الكافي وتعرضت حياة المرضى إلى الخطر. ويمكن تحسين فعالية العلاج بمضادات الفيروسات القهقرية عن طريق إيتائه كجزء من تعاون بحثي دولي حيث يمكن أن تساعد أوجه التعاون هذه في تحديد المشكلات وإيجاد الحلول وزيادة قدرة نظام الرعاية الصحية.

## 摘要

**问题:**

撒哈拉以南非洲引入艾滋病感染抗逆转录病毒治疗（ART）提高了数百万人的生活质量，降低了死亡率。然而，ART交付基础设施依然存在大量问题。

**方法:**

几内亚比绍艾滋病诊所的临床医生和研究人员通过建立临床数据库以及与国际研究人员合作识别ART交付的问题。

**当地状况:**

2007年，作为当地艾滋病医生和国际艾滋病研究人员之间的协作成立了比绍HIV队列组。在该国首都比绍的主要医院艾滋病诊所招募了患者。

**相关变化:**

2005年至2013年间，诊所治疗了5514名艾滋病毒阳性患者。当地卫生保健工作者和国际研究人员合作确定了影响ART交付的主要问题：药物供应不足；患者失访；实验室服务不足。设计了这些问题的解决方案。协作鼓励当地医生启动自己的研究项目以找到解决诊所问题的可能方案。

**经验教训:**

比绍艾滋病诊所面临提供足够高质量ART的众多障碍，病人的生命处于危险的境地。可以通过将ART作为国际科研合作组成部分交付来增强其有效性，因为这种合作有助于发现问题、找到解决方案和提高卫生保健系统的能力。

## Резюме

**Проблема:**

Внедрение практики антиретровирусной терапии (АРТ) для лечения ВИЧ-инфекции в странах Африки южнее Сахары позволило улучшить качество жизни миллионов людей и добиться снижения смертности. Тем не менее, остаются существенные проблемы, связанные с инфраструктурой для проведения АРТ.

**Подход:**

Благодаря созданию клинической базы данных и сотрудничеству с международными исследовательскими организациями, клиницисты и исследователи клиники по лечению ВИЧ в Гвинее-Бисау выявили ряд проблем при проведении АРТ.

**Местные условия:**

В результате сотрудничества между местными врачами, занимающимися лечением ВИЧ, и международными исследователями ВИЧ в 2007 году в Бисау была создана группа когортного исследования ВИЧ. Пациенты были набраны из клиники по лечению ВИЧ главного госпиталя страны в столице Бисау.

**Осуществленные перемены:**

С 2005 по 2013 г. в клинике прошли лечение 5514 ВИЧ-положительных пациентов. Работая совместно, работники местных медицинских учреждений и международные исследователи выявили основные проблемы, влияющие на проведение АРТ, а именно: недостаточную обеспеченность лекарствами, потерю из виду пациентов для последующего наблюдения и несоответствующие лабораторные услуги. Для решения этих проблем был предложен ряд мер. Данное сотрудничество поощрило местных врачей к запуску своих собственных исследовательских проектов по возможному решению проблем в клинике.

**Выводы:**

Клиника по лечению ВИЧ в Бисау столкнулась с многочисленными препятствиями при проведении АРТ на достаточно высоком уровне, что поставило жизнь пациентов под угрозу. Эффективность АРТ может быть повышена путем проведения терапии в рамках международного научного сотрудничества, поскольку такая совместная работа может способствовать выявлению проблем, нахождению решений и увеличению возможностей системы здравоохранения.

## Introduction

In sub-Saharan Africa, the introduction of antiretroviral therapy (ART) for patients with human immunodeficiency virus (HIV) infections has improved the lives of millions of people and decreased mortality.^1^ However, despite support from the Global Fund to Fight AIDS, Tuberculosis and Malaria and other donor organizations, the infrastructure for delivering ART in low-resource settings is still affected by substantial problems.^2^ Earlier diagnosis and more aggressive treatment of opportunistic disease could decrease mortality beyond that achieved by ART alone.^3^ Moreover, as the use of ART has increased, there have been reports of drug stocks running out because of insufficient human resources or poor infrastructure. In addition, frequently the means of monitoring the effects and side-effects of ART are not available.

The aim of this article was to reflect on the challenges faced in the field at an HIV clinic in Guinea-Bissau. Principally, we wanted to describe how an international research partnership helped identify clinical problems and find solutions while, at the same time, building the capacity of the health-care system to deal with an HIV epidemic in a vulnerable country.

## Local setting

Guinea-Bissau is located in western Africa and is one of the poorest countries on the continent.^4^ It gained independence from Portugal in 1974 after a war of liberation that caused tremendous damage to the country’s economic infrastructure. Since then, it has experienced considerable political and military upheaval. Unlike most countries in the subregion, Guinea-Bissau has experienced an increase in the spread of HIV-1 infection in recent years. In 1989, the country had the highest prevalence of HIV-2 infection ever reported whereas HIV-1 infection was nonexistent. However, the prevalence of HIV-2 infection is now decreasing, while that of HIV-1 infection is on the rise.^5^^,^^6^

In 2005, a national HIV programme was implemented in Guinea-Bissau by the Ministry of Health. However, it was only during 2007 that the programme led to an increase in the number of patients being treated. The Bissau HIV cohort study group was established in 2007 by the Bandim Health Project in Guinea-Bissau and Aarhus University Hospital in Denmark in collaboration with nurses and physicians from the Hospital Nacional Simão Mendes, which is Guinea-Bissau’s main hospital and is located in the capital Bissau. The Bandim Health Project is a member of INDEPTH, which is a network of 42 demographic surveillance system field sites in 20 countries in Africa and Asia.^7^ Since 1978, a demographic surveillance system established in Bissau by the Bandim Health Project has generated population and health data at the household level as part of a collaboration between the Ministry of Health in Guinea-Bissau and the Statens Serum Institut in Denmark. All patients with HIV infections who attended the HIV clinic at Hospital Nacional Simão Mendes were eligible for inclusion in the Bissau HIV cohort. The cohort study group created a database for all patients in the cohort and set up a biobank where blood samples from these patients were stored for use in research. The purpose of the database and the biobank was to help study how clinical, virological and immunological parameters influence the effectiveness of therapy.

Following the establishment of the Bissau HIV cohort study group, two doctoral students and 10 master’s students from Denmark, Iceland and Spain have worked at the HIV clinic for one to two years each and senior Danish researchers have visited on a regular basis. The close collaboration at the clinic between local HIV physicians and international HIV researchers provided a unique opportunity for sharing experience. The study group is supported financially and scientifically by the International Epidemiologic Databases to Evaluate AIDS network and the West African Platform for HIV Intervention Research. In addition, Danish universities and several Danish funding organizations have also provided financial support for research activities, including salaries for local staff and international researchers. Consequently, the study group is involved in collaborations both between developed and developing countries and between developing countries in Africa.

## Relevant changes

Between June 2005 and June 2013, 5514 patients older than 15 years were diagnosed with HIV infections and were offered care at the HIV clinic at the Hospital Nacional Simão Mendes (Table 1). All medical consultations, laboratory investigations and treatment were free of charge. Information on these patients was stored in the clinical database created by the Bissau HIV cohort study group and blood samples were stored in the associated biobank.

**Table 1:** Patients attending an HIV clinic, Guinea-Bissau, 2005–2013

Characteristic	No. (%)^a^ of patients (*n* = 5514)
**Sex**	
Female	3590 (65)
Male	1922 (35)
Missing data	2 (0.04)
**Age at first visit in years, median (IQR)**	36 (29–45)
**Type of HIV infection**	
HIV-1	3697 (67)
HIV-2	954 (17)
HIV-1 and HIV-2	598 (11)
Missing data	265 (5)
**Included in the Bissau HIV study cohort**	
Yes	3699 (67)
No	1815 (33)
**Baseline CD4+ T-lymphocyte count in cells/μL, median (IQR)**	197 (79–375)
**Started on ART**	
Yes	3170 (57)
No	2344 (43)

ART: antiretroviral therapy; CD4: cluster of differentiation 4; HIV: human immunodeficiency virus; IQR: interquartile range.

^a^ All values represent absolute numbers and percentages unless otherwise stated.

The delivery of ART involved a multitude of challenges at the clinic; these were identified during daily clinical work and routine data entry into the database as well as during ongoing research projects. These problems, their effects and proposed solutions are presented in Table 2. Local staff had heavy workloads and many of these problems would not have been identified in the absence of collaborative research with organizations in other countries.

**Table 2:** Problems with ART delivery at an HIV clinic, Guinea-Bissau, 2005–2013

Problem	Effect	Solution
**Inadequate drug supply**	Patients with a high CD4+ T-lymphocyte count experienced Stevens–Johnson syndrome on switching from efavirenz to nevirapine after stocks of efavirenz ran out;^8^ development of drug resistance due to treatment interruptions	Improve stock management, increase investment in health-care infrastructure and capacity
**Clinic relocation**	Patients lost to follow-up	Increase the focus on HIV infection at the hospital to give the disease a higher priority among policy-makers
**Widespread loss to follow-up**	Patients not adequately treated	Identify risk factors for patients being lost to follow-up so that effort can be focused on the most vulnerable;^9^ introduce educational activities for patients to improve health literacy; telephone patients who are late for appointments; visit patients lost to follow-up at home
**Poor treatment adherence**	Treatment failure and drug resistance	Identify risk factors for poor adherence;^10^ improve health literacy
**Laboratory inadequacies**		
Inadequate validation of HIV rapid tests	Errors in discriminating between infection with HIV-1, HIV-2 and both HIV-1 and HIV-2 occurred with the SD Bioline HIV 1/2 3.0 rapid test (Standard Diagnostics Inc., Yongin, Republic of Korea);^11^ ineffective treatment for HIV-2 infection using non-nucleotide reverse transcriptase inhibitors; expensive treatment for HIV-1 infection using protease inhibitors	Use other rapid HIV diagnostic tests
Temporary unavailability of biochemical tests and CD4+ T-cell count measurements	Delayed initiation of ART; late diagnosis of treatment failure; adverse events not diagnosed	Increase awareness of possible treatment failure
No HIV-RNA monitoring	Late diagnosis of treatment failure; development of drug resistance	Increase the ability of the laboratory to perform HIV-RNA measurements
Insufficient tuberculosis screening	Tuberculosis not diagnosed, leading to no tuberculosis treatment and increased mortality; no detection of drug-resistant tuberculosis	Introduce a simple clinical tuberculosis score together with a rapid urine test for the disease; introduce tuberculosis culture and drug-resistance tests
Insufficient hepatitis screening	No hepatitis treatment due to low sensitivity of rapid tests for hepatitis B and C viruses^12^	Increase awareness of the limitations of rapid tests

ART: antiretroviral therapy; CD4: cluster of differentiation 4; HIV: human immunodeficiency virus; RNA: ribonucleic acid.

Subsequently, awareness of these problems led to additional collaborative research projects between local HIV physicians and international researchers that aimed to explore possible solutions. In addition, the collaborations have encouraged local physicians to start their own research projects to find possible solutions to problems at the clinic. As a result, courses on good clinical practice, good laboratory practice and data management have been implemented and staff have taken part in English language lessons. Throughout, it was important to ensure that training for local staff was individualized. Furthermore, the synergies inherent in the rich spectrum of parties involved in research meant that knowledge and insights were multiplied.

## Discussion

The largest HIV clinic in Guinea-Bissau faced numerous obstacles in delivering ART at a sufficiently high quality and, as a result, patients’ lives were put in jeopardy. These difficulties may have been exacerbated by the frequent recurrence of political instability in the country. If similar issues are faced by the many ART facilities in Africa that report few data, it is likely that the implementation of ART in affected areas will be impaired. Moreover, there will also be a risk of publication bias since the clinics discussed in scientific publications may not be representative of the real situation in many areas. Previous studies have shown that there is little collaboration between researchers within developing areas and that most research on HIV is carried out in the developed world.^13^ Consequently, it is increasingly recognized that international collaborative research is important for tackling global public health problems. In particular, international partnerships, especially those between developed and developing nations, are necessary in the fight against diseases that are endemic in, or disproportionably affect, the developing world. As summarized in Box 1: (i) we identified a range of persistent problems affecting ART delivery in Guinea-Bissau that involved drug supply, patient retention and inadequate laboratory facilities; (ii) we believe that underreporting of experience with ART in similar clinics in Africa may lead to publication bias; and (iii) we observed that international collaboration is important for identifying health-care problems and devising solutions.

Box 1 **Summary of main lessons learnt**

- In Guinea-Bissau, there were substantial, persistent problems with the delivery of ART, due to inadequate drug supplies, loss of patients to follow-up and inadequate laboratory services.
- The occurrence of similar problems at the many ART facilities in Africa that report few data could impede the implementation of ART and result in publication bias.
- International research collaborations between high- and low-resource settings can help identify problems, find solutions and enhance the capacity of health-care systems to manage HIV infection.

Partnerships between academic institutions in developed and developing countries is important for the delivery of health care as well as for research and training. Fortunately, the size and range of such partnerships have increased recently.^14^ The Swiss Commission for Research Partnerships with Developing Countries has developed *A Guide for Transboundary Research Partnerships* as an aid to the establishment of academic partnerships with developing countries.^15^ Also, Guinea-Bissau has taken part in international collaborations for many years through the INDEPTH network.^7^

Our experience demonstrates that collaboration between physicians in high- and low-resource settings and between clinicians and researchers can help solve everyday clinical problems and enhance the capacity of the health-care system. Consequently, we believe that international research collaboration can help improve the effectiveness of ART in low-income countries and can benefit both partners. One unique facet of the collaboration in Guinea-Bissau was that researchers from developed countries lived in Guinea-Bissau and, as a result, developed a clear understanding of the problems faced in daily practice. In addition, the fact that we were able to follow up the large number of subjects in our HIV cohort for seven years despite difficult working conditions indicates that collaboration can be sustainable. An increasing number of scientific publications have resulted and it is hoped that additional funding for the cohort study group will further improve the capacity of the health-care system.

In conclusion, the management of people with HIV infection in vulnerable countries is still very challenging. However, international research collaboration can help identify problems and solutions, as well as enhance the capacity of the health-care system. Future research by the Bissau HIV cohort study group will demonstrate whether our identification of problems with the delivery of ART has led to measurable benefits, such as fewer patients being lost to follow-up, lower mortality, better diagnosis of opportunistic infection, more frequent detection of treatment failure and better-educated local staff.
